# The application of composite scaffold materials based on decellularized vascular matrix in tissue engineering: a review

**DOI:** 10.1186/s12938-023-01120-z

**Published:** 2023-06-19

**Authors:** Jingying Li, Xiao Chen, Miaoling Hu, Jian Wei, Minhai Nie, Jiana Chen, Xuqian Liu

**Affiliations:** 1 Department of Periodontics & Oral Mucosal Diseases, The Affiliated Stomatology Hospital of Southwest Medical University, Luzhuo, 646000 China; 2 Oral & Maxillofacial Reconstruction and Regeneration of Luzhou Key Laboratory, Luzhou, 646000 China; 3 Department of Stomatology Technology, School of Medical Technology, Sichuan College of Traditional Medicine, Mianyang, 621000 China; 4 Department of Orthodontics, Mianyang Stomatological Hospital, Mianyang, 621000 China

**Keywords:** Decellularized vascular matrix, Extracellular matrix, Vascular graft, Vascular scaffold

## Abstract

Decellularized vascular matrix is a natural polymeric biomaterial that comes from arteries or veins which are removed the cellular contents by physical, chemical and enzymatic means, leaving only the cytoskeletal structure and extracellular matrix to achieve cell adhesion, proliferation and differentiation and creating a suitable microenvironment for their growth. In recent years, the decellularized vascular matrix has attracted much attention in the field of tissue repair and regenerative medicine due to its remarkable cytocompatibility, biodegradability and ability to induce tissue regeneration. Firstly, this review introduces its basic properties and preparation methods; then, it focuses on the application and research of composite scaffold materials based on decellularized vascular matrix in vascular tissue engineering in terms of current in vitro and in vivo studies, and briefly outlines its applications in other tissue engineering fields; finally, it looks into the advantages and drawbacks to be overcome in the application of decellularized vascular matrix materials. In conclusion, as a new bioactive material for building engineered tissue and repairing tissue defects, decellularized vascular matrix will be widely applied in prospect.

## Background

Atherosclerosis and heart disease remain important causes of morbidity and mortality worldwide, and the treatment of coronary and peripheral vascular disease often requires the replacement of damaged vessels with vascular grafts. For the reconstruction of large artery, such as the aorta or iliac artery, grafts made of expanded polytetrafluoroethylene (EPTFE) or polyester are commercially available with satisfactory results currently. However, due to the inherent properties of synthetic materials, synthetic grafts are not suitable for reconstructing smaller diameter (< 5 mm ID) arteries, and the leading causes of graft failure are thrombosis, limited re-endothelialization, and neointimal hyperplasia [1]. Autologous and allogeneic vessels are ideal vascular substitutes. However, the supply of autologous vascular often fails to satisfy the clinical needs due to the secondary injury of donor and the limitation of materials. The allogeneic vascular are convenient to harvest,  but their application is limited by immunological rejection and secondary infection.

**Fig. 1:**
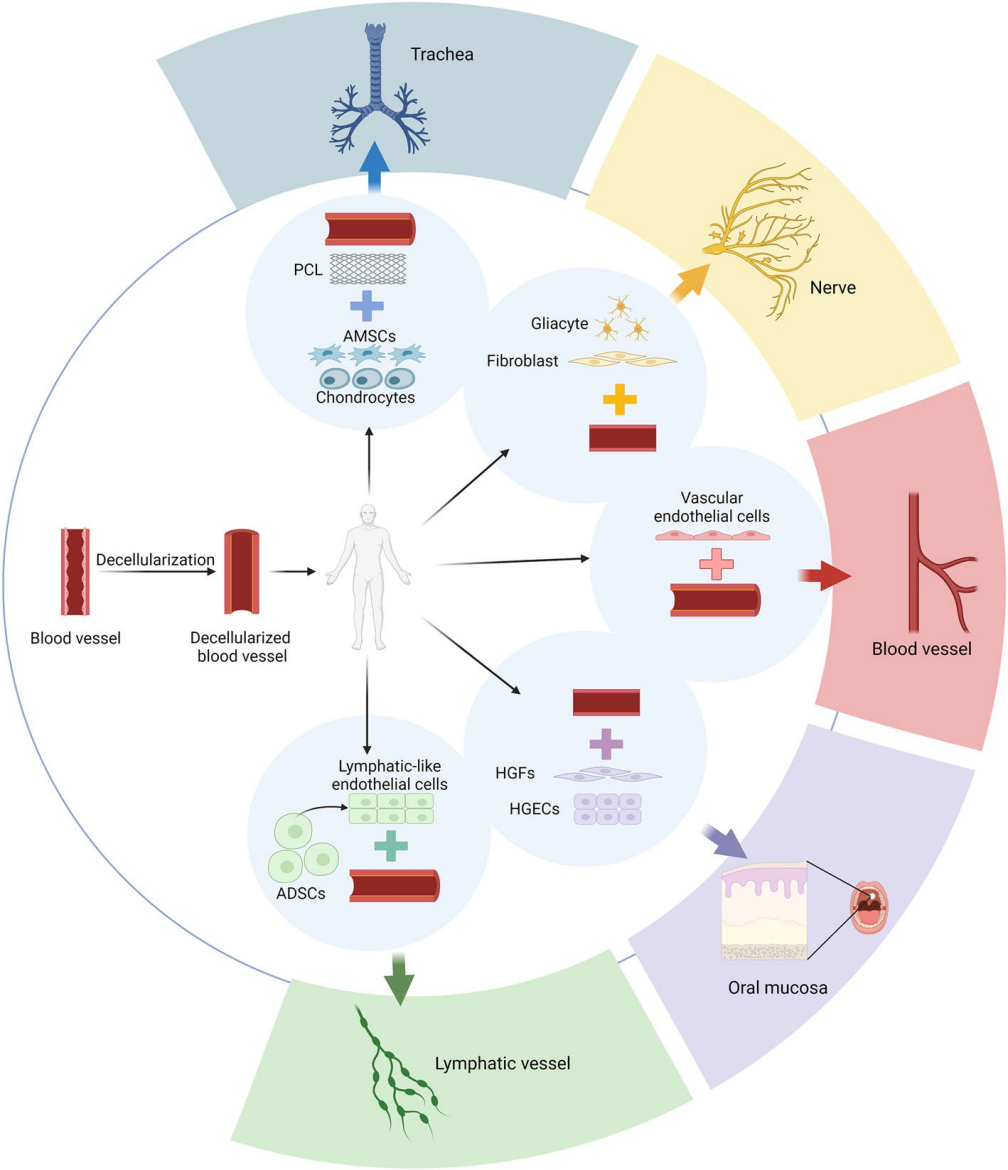
The application of decellularized vascular matrix in tissue engineering. Created with BioRender.com

The development of tissue engineering and biomaterials has provided new ideas to solve the above problems. One of the most promising approaches is the use of decellularized tissue as scaffold material [2–4]. Because the scaffold material of natural biological origin is rich in various growth factors, matricellular proteins and bioactive vesicles after decellularization, it still has the function of activating endogenous tissue repair [5]. Moreover, the preserved extracellular matrix (ECM) can regulate cellular physiological activities and functions [6]. At the same time, the decellularized bioscaffold also reduces the immunological rejection and post-transplant calcification rates associated with natural biological tissues to a great extent [7]. The use of decellularized vascular matrix as a scaffold for the repair and reconstruction of tissue defects can overcome the immunological rejection of the organism to materials of xenogeneic origin and reduce various complications in the later stages of surgery. Currently, the research and application of decellularized vascular matrix are mainly in vascular tissue engineering and soft tissue engineering, and the research on other tissue engineering is limited. This review summarizes the progress of the application of composite scaffold materials based on decellularized vascular matrix in tissue engineering research. Part of application of decellularized vascular matrix in tissue engineering is shown in Fig. 1.

## Characterization and preparation

Decellularized vascular matrix is a biological scaffold material that comes from blood vessels of biological origin, which is removed immunogenic components by chemical, physical or other methods. It retains the original three-dimensional spatial structure as well as functional matrix proteins for cell attachment, proliferation and differentiation, with the function of transmitting physical, chemical and biological signals [8]. After the decellular treatment, the ECM and cytoskeletal proteins of the blood vessels should be retained, while the immunogenic substances of the cells, such as DNA [9], MHC I-complexes, and MHC II-complexes [10], should be removed as far as possible. The remaining ECM components mainly include: glycoproteins (fibronectin, laminin, collagen), proteoglycans (heparin, chondroitin sulfate) and elastin, which constitute a complex three-dimensional network. These molecules have good biocompatibility and can effectively promote the adhesion and proliferation of cells. Among them, collagen is the main component of the basement membrane, which provides a fibrous protein matrix, and the water permeability and water absorption of it are high. In addition, collagen and elastin have abundant RGD (Arg-Gly-Asp) sequences, vascular endothelial cells (VECs) and vascular smooth muscle cells (VSMCs) bind to RGD sequences by integrins (α1β1, α2β1), thus adhere to vascular lumen [11, 12]. Through proteomic analysis, scholars have identified the protein components of the decellularized vascular matrix, which is rich in collagen [13–15]. Table 1 presents the species of collagen in decellularized vascular matrix prepared by different decellularization methods.

**Table 1:** List of representative identified collagens in decellularized vascular

Tissue	Decellularization methods	Collagens	Refs.
Human umbilical artery	CHAPS/EDTA/SDS	Collagen VI	[13]
		Collagen XII	
		Collagen XIV	
		Collagen XVIII	
Human aorta	EDTA/SDS	Collagen I	[14]
		Collagen IV	
		Collagen V	
		Collagen VI	
		Collagen VIII	
		Collagen XII	
		Collagen XIV	
		Collagen XV	
		Collagen XVIII	
Human renal artery	SDS/Triton X-100/EDTA	Collagen I	[15]
		Collagen III	
		Collagen IV	
		Collagen V	
		Collagen VI	
		Collagen VIII	
		Collagen XII	
		Collagen XIV	
		Collagen XV	
		Collagen XV	
		Collagen XXI	

### Physical methods

The physical methods of decellularization refer to promoting the destruction and dissolution of cell by adjusting temperature, force, and pressure, etc. Physical methods include freeze-thawing, high hydrostatic pressure treatment, and perfusion-decellularization.

#### Freeze-thawing

The formation of intracellular ice crystals at low temperatures (−80℃) destroys cell membranes and leads to the release of cell contents. Then, the cell is melted at room temperature, and the cell structure is broken by repeated freezing and thawing, so as to achieve the purpose of decellularization. Multiple freeze-thaw cycles can be used during decellularization and do not significantly increase the loss of ECM proteins in tissues [16, 17]. However, freeze-thawing cannot completely remove the immunogenicity of the cell matrix, and other methods are needed to further remove the residual components of cells in the tissue, for example, combining with detergents [18].

#### High hydrostatic pressure treatment

Applying a pressure greater than 600 MPa to disrupt the cell membrane can eliminate or reduce the exposure time of irritating detergents in the process of tissue decellularization. At controlled temperatures, Funamoto obtained decellularized pig blood vessel by immersing it in saline and subsequently exposing it to increasing pressures up to 980 Mpa [19]. It was found that the collagen fiber layer was dense and relatively complete, and in vitro thrombus formation time experiments demonstrated a superior antithrombotic ability. In their subsequent in vivo studies, allogeneic acellular vessels treated with high hydrostatic pressure showed 100% patency within 4 weeks; and they observed vessel lumen was covered by VECs [20]. Moreover, they observed that after decellularization with high hydrostatic pressure, washing at 4 °C was beneficial for protection of collagen fibers and structures of vascular [21].

#### Perfusion-decellularization

Perfusion-decellularization utilizes endogenous vascular channels to deliver decellularized solvent to tissues with high density, which, importantly, allows the generation of decellularized scaffolds from whole organs and complex tissues [22, 23]. In study of Eyre, perfusion-decellularization with sodium hydroxide solution used to remove cellular components, while preserving structural and mechanical integrity and significantly supporting the adhesion of human umbilical vein endothelial cells [24].

### Chemical methods

Chemical methods include the use of acids, alkalis, detergents, alcohols and other solvents, and the most commonly used chemical method is detergent decellularization. Detergents can be divided into ionic, nonionic and zwitterionic detergent.

#### Ionic detergent

Ionic detergents are effective in lysing cell membranes and separating DNA from proteins, but they can easily damage ECM proteins. Sodium dodecyl sulfate (SDS) and sodium deoxycholate (SD) are commonly used ionic detergents. Bertanha compared the effects of 2% SD and 1% SDS on rabbit vena cava and found that SDS significantly disrupted intravascular collagen and microstructure [25]. In the preparation of decellularized vessels, SDS was used at concentrations ranging from 0.1% to 1%. As concentration and decellularization time increased, the clearance of cells and damage to ECM become more significant. Low concentration of SDS can effectively remove cells from vein without significantly damaging the ECM [26].

#### *Nonionic detergen*t

The nonionic detergent Triton X-100  is commonly used to prepare decellularized vascular scaffolds. Dahl compared the effects of three decellularization methods on porcine carotid arteries and showed that Triton X-100 alone was ineffective in removing nucleic acid of the arteries, thus the decellularization effect was not ideal [27]. Triton X-100 is weak in removing proteins and therefore has less damage to the ECM and protein-based bioactive factors, facilitating cell adhesion and growth on the surface of the scaffolds.

#### Zwitterionic detergent

Zwitterionic detergents include SB-10, SB-16 and 3-[(3-cholamidopropyl) dimethylammonio]-1-propanesulfonate (CHAPS). CHAPS is commonly used in vascular decellularization. The effect of CHAPS is relatively modest compared to the ionic detergent SDS, the CHAPS-decellularized tissue retained more collagen, glycosaminoglycan and elastin, while removing 95% of the nucleic acid [28, 29]. However, when compared with the nonionic detergent Triton X-100, CHAPS causes greater structural disruption of ECM, which is not conducive to the proliferation and adhesion of VECs during recellularization [30].

### Biological methods

Biological decellularization protocols mainly involve enzymatic reactions, usually refers to proteases and nucleases. Trypsin selectively cleaves cell adhesion proteins on the carboxyl side of arginine or lysine to detach cells from the tissue surface, which can disrupt the ECM surrounding collagen fibers, create tiny channels, and facilitate subsequent penetration of decellularized solvent. Trypsin is time-dependent in the removal of cellular and ECM components, and 24 h of exposure is sufficient to cause irreparable damage to the ECM [31, 32]. DNase and RNases are endonuclease enzymes that hydrolyze the DNA strand and RNA strand, respectively, and can be added to detergent treatment to help remove residual DNA if effective decellularization cannot be achieved with detergents alone [32]. Continuous enzymatic digestion using trypsin, DNase and RNase can also achieve better decellularization effect, and ECs can form a continuous cell layer on the surface of the vascular scaffold [33].

**Fig. 2:**
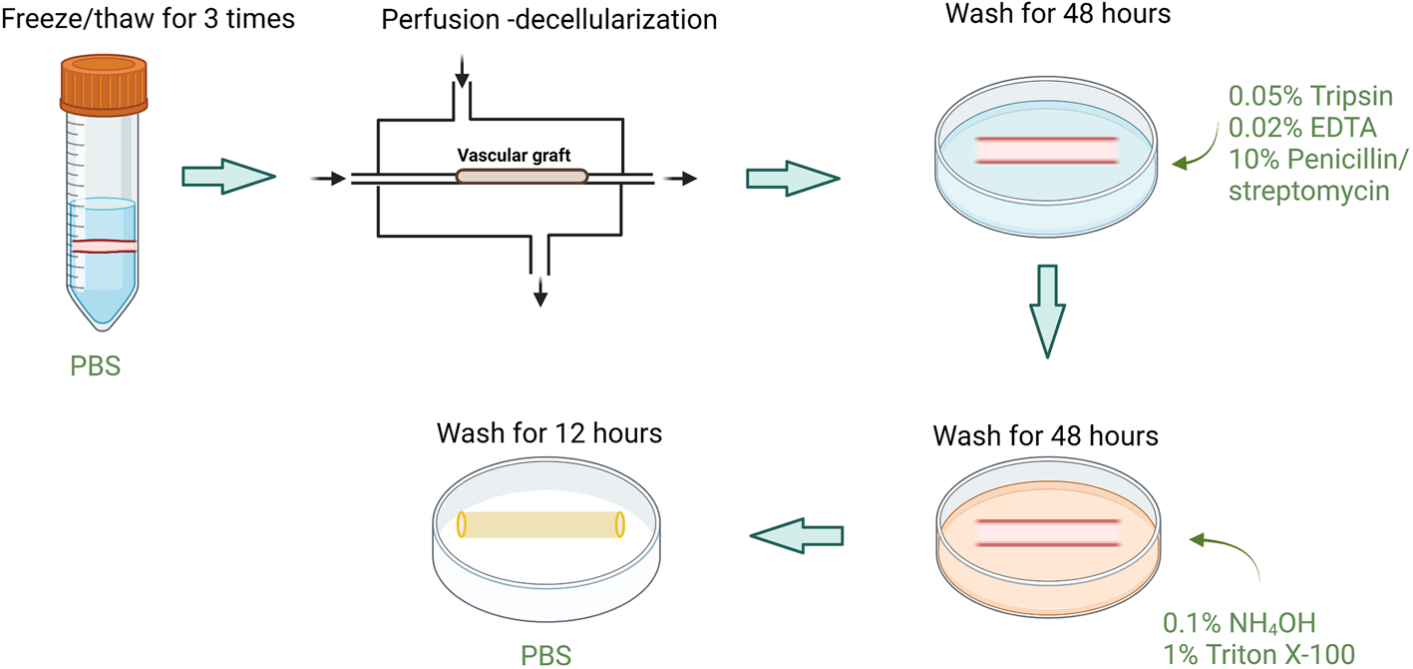
The combination of multiple decellularization methods used in the study of Ilanlou. Created with BioRender.com

In summary, no matter what kind of decellularization method, there are advantages and limitations as shown in Table 2**.** The key criterion is removal of cellular components and retain of ECM structure, biological activity and mechanical properties. Therefore, in order to obtain the optimal balance of removing the cells and retaining the ECM, scholars often combine a variety of decellularization methods. Table 3 demonstrates combination of different decellularization method and results. Figure 2 shows the combination of multiple decellularization methods used in the study of Ilanlou [53].

**Table 2:** Summary of result of different decellularization methods

	Residual DNA	ECM structure	Thrombus formation time	Cell behavior	Refs.
Triton X-100	< 0.5 ng/mg	Maintained	Without evaluation	High ECs viability	[30]
SDS	< 0.5 ng/mg	Maintained		Poor ECs viability	
SD	< 1 ng/mg	Maintained		Poor ECs viability	
CHAPS	< 0.5 ng/mg	Maintained		Poor ECs viability	
Enzymatic digestion	< 0.1%	Intact		Forming a complete, intact ECs layer	[33]
High hydrostatic pressure	small(< 50 ng/mg)	Maintained	Until 15 min	Cell adhesion was similar to TCPS	[16]
Freeze-thawing method	small(< 150 ng/mg)	Maintained	Within 8 min	Cell adhesion decreased during the 7 days cultivation	
Perfusion	24.7 ± 1.7 µg/mg	Retained	Without evaluation	Forming a confluent ECs monolayer	[24]

**Table 3:** The results of different decellularization methods for vascular scaffold in vitro or in vivo

Decellularization method	Tissue	Experimental model	In vitro or in vivo results	Refs.
Triton X-100 and trypsin	Bovine jugular vein	Rat	Reduced platelet adhesion, stimulated proliferation of ECs in vitro, and reduced calcification in vivo	[51]
Triton X-100, RNase, and DNase	Porcine femoral artery	Rat	ECs and myofibroblasts were detectable within 1 month, 97.3% patency rate in 6 months	[43]
Triton X-100, SD, RNase and DNase	Rat infrarenal abdominal aorta	Rat	After modified with GCSF, observed superior cellular and ultrastructural preservation	[59]
Triton X-100, trypsin, RNase, and DNase	Rabbit abdominal aorta	Dog	After modified with heparin, bFGF, and VEGF 145, patency rate was 100% at 1, 3, and 9 months	[58]
Freeze‐thawing and SDS	Porcine aorta	Rat	Less calcification and adverse inflammatory response, enhanced ingrowth of myofibroblasts and ECs	[17]
Freeze‐thawing, Triton X-100 and SDS	Porcine carotid artery	In vitro experiment only	Well-preserved composition, structure, and mechanical properties	[18]
Perfusion and Triton X-100	Sheep carotid artery	In vitro experiment only	Completely removed cell nuclei and preserved three-dimensional structure and mechanical properties of native tissue	[53]
	Placental and umbilical cord artery	In vitro experiment only	Excellent biocompatibility and mechanical properties	[73]
High hydrostatic pressure and DNase	Porcine radial artery	Rat	100% patency rate and without thrombosis in 2 weeks, ECs were found to cover luminal surfaces	[20]

## Recellularization

Before implantation of decellularized vascular scaffolds, recellularization and adhesion of functional endothelial cells (ECs) play a crucial role in maintaining patency. Recellularization in vitro with autologous cells has been reported to improve patency rate [34, 35], as well as reduce neointimal hyperplasia [35] and local inflammatory response [36], thus significantly improving performance of vascular scaffolds. However, recellularization before implantation takes a long time, so patients undergoing emergency surgery cannot wait; the recellularization process also increases the risk of scaffolds contamination; on the other hand, the optimal cell source for autologous endothelialization in vitro has not been identified. Although endothelial progenitor cells (EPCs) have been proposed as a suitable source of cells, collecting EPCs from peripheral blood is extremely inefficient; what’s more, purification and culture of EPCs are difficult [37].

However, the collagen on the surface of scaffolds will be exposed if not recellularized before implantation, which may lead to thrombosis and vascular occlusion in the initial phases [38]. Due to the complexity of in vitro tissue engineering techniques, tissue engineering in situ or so-called guided tissue regeneration came into being. This approach is defined as coating the scaffold with homing factors to induce endothelialization in vivo and using the inherent homing ability of bone marrow mesenchymal stem cells (MSCs) in blood circulation. Therefore, many investigators have modified vascular scaffolds with bioproteins and growth factors to recruit EPCs [39]. EPCs are able to differentiate into VECs and VSMCs in a specific microenvironment to promote recellularization in vivo. Yamanaka H used rat tail artery as a novel scaffold material for vascular tissue engineering, analyzed the denaturation of ECM during decellularization or peptide modification and the stability of peptides in the lumen of the scaffold, and reported the possibility of in vivo recellularization of decellularized tissue [40]. Recellularization of the decellularized vascular scaffolds in vivo not only facilitate vascular tissue reconstruction, but may also prevent thrombosis and maintain the patency of the vascular grafts.

## Application in vascular tissue engineering

Cardiovascular diseases and various vascular-related diseases pose a significant threat to human health, and vascular grafting and reconstruction are the primary means of treatment. In order to provide better clinical treatment solutions and achieve more effective surgical results, the application of vascular graft materials in tissue engineering has been gradually studied. Decellularized vascular scaffold materials are mainly used in vascular tissue engineering for repair and reconstruction, and are an ideal alternative for autologous vascular grafts. Early endothelialization and inhibition of thrombosis are critical steps in the success of vascular grafts. Researchers have significantly improved the performance of decellularized vascular scaffolds by combining biomolecules, cell adhesion peptides, growth factors and degradable synthetic polymers with decellularized vascular scaffolds to form composite scaffolds as shown in Table 4.

**Table 4:** Surface modified decellularized vascular matrix for vascular scaffolds

Tissue source	Animal model	Modification	Patency rate	Refs.
Human placenta chorion	Rat infrarenal aorta	Heparin	100% at 1 month	[50]
Bovine jugular vein	Rat	Heparin/DHI	–	[51]
Sheep carotid artery	–	CKC	–	[53]
Rat abdominal aorta	Rat abdominal aorta	S1P	100% at 2 weeks	[55]
Rabbit abdominal aorta	Dog femoral artery	VEGF/bFGF	90% at 18 months	[58]
Rat abdominal aorta	Rat abdominal aorta	GCSF	100% at 8 weeks	[59]
Rat thoracic aorta	Rat infrarenal abdominal aorta	HG-VEGF	100% at 8 weeks	[39]
Porcine aortic valve	Rat	VEGF /PLC	–	[60]
Rat abdominal aorta	–	CBP/heparin	–	[61]
Equine carotid artery	Sheep right cervical AV fistulae	CCN1	100% at 14 weeks	[65]
Rabbit artery	Nude mice	HLC-I	–	[67]
Rat aortic conduits	Rat aortic conduit	Fibronectin	–	[68]
Rat thoracic aorta	Rat infrarenal aorta	Fibronectin/ SDF1α	100% at 8 weeks	[69]
Bovine pericardium	Nude rat abdominal aorta	PPF	100% at 2 weeks	[71]
Umbilical cord and placental artery	–	GO	–	[73]
Rat abdominal aorta	–	POC	–	[74]
Rat descending aorta	Rat abdominal aorta	PCL	100% at 6 weeks	[77]

### Decellularized vascular scaffold materials modified with antithrombotic molecules

A potential solution to prevent thrombosis and graft rejection is the surface modification of vascular grafts with antithrombotic molecules. Heparin is named for it was first found in liver, which is a negatively charged natural anionic polysaccharide and a highly sulfated glycosaminoglycan. It has a strong anticoagulant effect by activating antithrombin II and inhibiting the coagulation cascade to prevent thrombosis. As a common clinical anticoagulant and antithrombotic drug, heparin also has a variety of biological activities such as anti-intimal hyperplasia, selective adsorption of plasma proteins and anti-blood plate aggregation, which is often used on the surface of acellular heart valves to induce endothelialization through the interaction of endothelial growth factor receptors with ECs, prevent platelet adhesion, and inhibit intimal hyperplasia caused by proliferation of VSMCs [41, 42]. It has been shown in many studies that small-diameter vascular grafts exhibit excellent antithrombogenicity, mechanical property, and biocompatibility by heparinization [43–49]. Schneider decellularized human placental chorionic villi as the source of small-diameter vascular grafts, then cross-linked them with heparin. Biocompatibility was tested by culturing the scaffolds with primary human macrophages in vitro and implanting the scaffolds into the infrarenal aorta of SD rats in vivo. The modified scaffolds showed good biocompatibility, low immunogenicity, high patency rate, and no sign of thrombosis or aneurysm formation [50]. Tao prepared heparin nanomodified decellularized bovine jugular vein scaffolds by self-assembling alternating linkage of heparin and dihydroxyl-iron (DHI), and evaluated the properties of the scaffolds in vitro and in vivo. After sustained release of heparin for several weeks in vitro, the biomechanical stability of the scaffolds was significantly enhanced. Importantly, after implanting in a rat model subcutaneously, the modified scaffolds showed to significantly reduce platelet adhesion, stimulate proliferation of ECs, reduce calcification, and enhance biomechanical stability [51].

Although heparinized vascular scaffolds are effective in preventing thrombosis, some clinical studies have found that heparin may cause side effects such as thrombocytopenia, bleeding, heparin-associated osteoporosis, skin reactions, and eosinophilia in some cases, making it necessary to find other anticoagulants with fewer side effects [52]. In the study of Ilanlou, carboxymethyl κ carrageenan (CKC) was introduced as a novel anticoagulant in vascular tissue engineering. They found that CKC-modified scaffolds significantly reduced platelet adhesion, and supported ECs viability, proliferation, and nitric oxide production, which provided a promising solution for thrombosis in small-diameter vessels [53]. However, this experiment was not validated in vivo. Sphingosine-1-phosphate (S1P) has been shown to have antithrombotic and pro-angiogenic properties [54]. Hsia modified allogeneic vascular scaffolds with S1P. Due to increased proliferation and adhesion of VECs, rats implanted with S1P-coated re-endothelialized scaffolds exhibited 100% survival and patency within 2 weeks [55].

### Decellularized vascular scaffold materials modified with growth factors

Vascular endothelial growth factor (VEGF) promotes migration and proliferation of VECs [56], which plays an important role in angiogenesis. Numerous in vitro and in vivo studies have been performed in the past, showing their remarkable potential for promoting vascular growth [57], and their use to modify decellularized vascular scaffolds can enhance the endothelialization. Kong coated decellularized vascular scaffolds with heparin and then sequentially transplanted with basic fibroblast growth factor (bFGF) and VEGF. The physicochemical properties, in vivo anticoagulant activity, biocompatibility, and clinical feasibility of modified scaffolds were comprehensively evaluated. After implantation, there was no significant difference between the natural vessels and the heparinized decellularized vascular scaffolds containing VEGF145 and bFGF. The patency rate was 100% at 1, 3 and 9 months, and up to 90% at 18 months [58]. The scaffold is important for small-diameter vascular grafts in shortening surgical waiting time, reducing costs, and reducing the risk of in vitro infection. Granulocyte colony stimulating factor (GCSF) is a hematopoietic cytokine clinically used to mobilize progenitor cells in the bone marrow and increase their number in the circulation. Kang investigated the effect of GCSF on inhibiting poor vascular remodeling of small-diameter aortic conduits. This factor reduced adverse vascular remodeling by reducing intimal hyperplasia and enhancing endothelialization [59].

The slow and steady release of VEGF from vascular scaffolds is important for VEGF to work in vivo over the long term. In a study of Iijima, VEGF was combined with temperature-sensitive aliphatic polyester hydrogel (HG). Maintenance of the luminal HG-VEGF coating in vivo for up to 4 weeks was confirmed by rhodamine labeling, and Doppler ultrasound demonstrated the function of graft in vivo for up to 8 weeks. Compared with the control group, histological and immunohistochemical analysis of the grafts after 4 and 8 weeks in vivo showed a significant increase in endothelial formation in the HG-VEGF group [39]. In the study of Zhou, composite valves were prepared by encapsulating VEGF into polycaprolactone (PCL) nanoparticles and then introducing PCL nanoparticles into decellularized aortic valves. It showed a slow drug release rate, low hemolysis and anti-platelet adhesion ability, and a large number of capillaries formed in the composite valves after 8 weeks of subcutaneous implantation in rats [60].

### Decellularized vascular scaffold materials modified with bioactive macromolecule

In order to regulate cell attachment and proliferation of the artificial vascular grafts and prevent aneurysm formation, proteins, peptides, antibodies and more have been used to modify the decellularized vascular matrix. In the research of Jiang, a collagen-binding peptide (CBP) was covalently linked to heparin to form a heparin derivative (CBP-heparin), which was used to modify the vascular ECM. The result showed that modification of ECM with CBP-heparin led to increased deposition of functional heparin, subsequently reduced thrombogenicity and stabilized adhesion of ECs to the lumen [61]; however, this study had some drawbacks, such as aneurysm formation and lack of long-term follow-up. It has been shown that matricellular protein 1 (CCN1), a protein of the CCN family, can promote homing of ECs and EPCs, facilitate angiogenesis, and regulate inflammation [62–64]. Boer coated decellularized horse carotid arteries with CCN1 and evaluated its cytotoxic and angiogenic effects in vitro, assessed it in vivo cell regeneration, local biocompatibility, neovascularization and immunogenicity in a sheep model. The results revealed that the CCN1 coating produced a non-toxic matrix and did not affect fibroblast and ECs vitality; moreover, CCN1 coating reduces leukocyte infiltration and fibrosis and supports neovascularization [65]. The CCN1 coating of the vascular scaffold improves local biocompatibility and accelerates scaffold remodeling by enhancing cell regeneration and immune tolerance, making it a promising tool for the development of bioartificial vascular grafts. Liu presented a composite vascular scaffold, which was prepared by combining human-like collagen I (HLC-I) with acellular vascular matrix (ACVM), then performed a series of experiments to test the water absorption, biomechanics, compression resistance, cytotoxicity and ultrastructure of the composite vascular scaffolds compared with natural rabbit arteries. The result showed that the composite vascular scaffold performed similarly to natural rabbit arteries [66, 67]. Therefore, ACVM-0.25% HLC-I may be an ideal scaffold material for the construction of tissue-engineered vessels. Assmann implanted decellularized aortic catheters that coated with fibronectin on the surface into rats and found that fibronectin improved the cell adhesion and biocompatibility of decellularized vascular scaffold, leading to significantly faster endothelialization. However, the disadvantage is the aggravation of neointimal hyperplasia [68]. Sugimura et al. modified decellularized vascular scaffolds with the combination of fibronectin and stromal derived factor 1α (SDF1α) and observed similar results [69].

### Decellularized vascular scaffold materials modified with synthetic polymers

Vascular grafts made from synthetic polymers have disadvantages such as thrombosis, intimal hyperplasia, calcification, chronic inflammation and no growth potential. Although decellularized vascular scaffolds have good histocompatibility and antithrombotic properties, the decellularization process may disrupt biomechanics and accelerates the deformation and degradation of elastin, ultimately leading to vascular scaffolds expansion and even aneurysm formation. To address these issues, many researchers have combined synthetic polymers with decellularized small-diameter vessels to create hybrid tissue-engineered vascular scaffolds. Polypropylene fumarate (PPF) has shown promising results in vascular grafts, specifically its ability to maintain the mechanical properties of the pericardium and reduce the chronic inflammation associated with the natural bovine pericardium [70]. Kimicata combined decellularized extracellular matrix (dECM) with PPF. It was found that dECM + PPF scaffolds exhibited sufficient circumferential stress and rupture pressure in vitro, and suture retention was preserved in vivo; the modulus of dECM + PPF matched that of human coronary arteries and saphenous veins. It was showed endothelialization of vascular scaffolds and tissue growth in vivo [71]. In general, the dECM + PPF composite scaffold provides a robust solution to overcome the limitations of current therapeutic approaches for small-diameter vascular grafts.

Graphene oxide (GO) is a special two-dimensional nanomaterial. GO also has some unique chemical properties, such as large surface area, strong oxygen function, good electrical conductivity and good biocompatibility. These chemical properties lay the foundation for its biomedical applications in biomedical fields such as bioimaging, biosensing, drug carriers, and cryotherapy [72]. Pereira decellularized placental and umbilical cord arteries and perfused them with a suspension of GO. Compared to decellularized umbilical arteries, GO coating increased maximum force by 27%, the burst pressure by 29%, the strain by 25% and the compliance by 10%. The achieved theoretical burst pressure (1960 mmHg) and compliance (13.9%/100 mmHg) were similar to those of the human saphenous vein and mammary artery, respectively. In addition, GO coating did not impair adhesion of ECs, but reduced platelet and bacterial adhesion to decellularized arteries, making it a promising alternative to allogeneic grafts in coronary and peripheral bypass grafting [73]. Jiang combined decellularized rat aorta with a biodegradable and biocompatible elastomer poly (1,8 octane diol citrate) (POC, 1 wt.%). POC-ECM composite scaffold significantly reduced platelet adhesion and supported the adhesion of VECs and a small number of VSMCs in vitro [74]. However, this study lacked experimental validation in vivo. PCL is a biodegradable polymer material with excellent biocompatibility and mechanical properties. Some scholars electrostatically spun PCL nanofibers outside decellularized aortic vascular scaffolds, which significantly enhanced the biomechanics of decellularized vessels [75, 76]. In a study of Yang, rapamycin was incorporated into PCL, and the results showed that the outer layer of electrospun PCL effectively delivered rapamycin to the inner layer of decellularized rat aorta, which inhibited excessive proliferation of VSMCs and significantly reduced neointimal hyperplasia without impairing regenerative epithelialization and M2 macrophage polarization [77]. The combination of synthetic polymers with decellularized vascular matrix to construct composite scaffolds enabled the vascular scaffolds to retain excellent mechanical properties and biocompatibility, providing a new idea for tissue engineering of small vessel grafts.

### Photo-oxidative cross-linked decellularized vascular scaffold materials

Decellularization does not completely reduce the antigenicity of biological scaffolds, and appropriate collagen cross-linking methods can reduce the antigenicity of structural proteins, reduce immunological or foreign body response, and decrease tissue degradation. Photo-oxidative cross-linking is virtually non-cytotoxic and has chemical, enzymatical and in vivo stability [78, 79]. The basic principle of the oxidation method is that a variety of amino acids in biological tissues, such as tryptophan, histidine, tyrosine and methionine, are oxidized by visible light irradiation and cross-linked between molecules in the presence of suitable photosensitizers (methylene blue, rose Bengal dye). In the study of Lu, the performance of photo-oxidized cross-linked decellularized bovine jugular vein catheters in circulating implantation was evaluated through a dog RV-PA attachment model, and decellularized catheters were used as controls. Preliminary results supported that photo-oxidized cross-linked decellularized bovine jugular vein catheters can prevent calcification and thrombosis, with regenerative capacity and remarkable hemodynamic performance [80]. The anti-calcification properties of photo-oxidative cross-linked decellularized bovine jugular vein catheters were validated in their subsequent experiments [81]. Similar findings were reported by Pennel, Wang [82, 83].

In the study of Schneider, riboflavin-mediated ultraviolet ray (UV) cross-linking was used to uniformly crosslink the collagenous ECM of the scaffolds. The characteristics and biocompatibility of the scaffolds with and without UV cross-linking were studied in vitro and in vivo. The mechanical strength and luminal surface smoothness of UV cross-linked decellularized vascular scaffolds were significantly improved. Cell seeding using human ECs showed no cytotoxic effect of UV cross-linking treatment. Short-term aortic implantation in rats showed cell migration and differentiation of host cells [84]. Thus, UV cross-linking is an effective way to improve the characteristics of decellularized vascular scaffolds. Liu used photo-oxidation and pentagalloyl glucose to crosslink decellularized vascular scaffold, then implanted it into rabbit abdominal aorta. After short-term aortic implantation in the rabbits, collagen regeneration and differentiation of host smooth muscle cells was observed. Due to remodeling and stabilization of the neointima, no occlusion or stenosis occurred and a good patency was maintained (100%). Biomechanical results showed improved compliance, suture retention and resistance to elastase degradation [85]. The limitations of this study are that the time of implantation in the rabbit abdominal aortic model was too short, long-term patency and remodeling still need further study, and small-diameter vascular grafts still need to be studied and observed in large animal models before clinical application.

## Applications in wound healing

Decellularized vascular matrix is rich in collagen, glycosaminoglycans (e.g., acetyl heparan sulfate), proteoglycans (e.g., perlecan), and glycoproteins, all of which are involved in the wound healing process. Researchers have used decellularized vascular matrix as a wound dressing, tested its cytocompatibility in vitro, and evaluated its effect on wound healing in animal experiments. As a result, it was observed that the dressing had good hemostatic properties, cytobiocompatibility and histocompatibility, and it promoted wound angiogenesis and reduces scar formation in vivo [86, 87].

## Applications in abdominal wall repair

Decellularized vascular matrix can also be used for abdominal wall repair [88–90]; however, it was found in some studies that this bio-derived material tended to degrade after implantation and the mechanical strength and tensile strength decreased with time goes by. In order to overcome these limitations, Nowacki [91] and Zhang [92] used autologous MSCs to achieve in vitro recellularization, which significantly improved their mechanical strength and function.

## Applications in trachea tissue engineering

Ghorbani combined decellularized rabbit aorta with electrospun PCL which was seeded with primary chondrocytes and adipose-derived mesenchymal stem cells to construct a composite scaffold, then implanted to replace the trachea allogeneically. The composite scaffold was observed to be suitable for tracheal tissue engineering in terms of lumen morphology, mechanical properties, biocompatibility, and cell adhesion [93].

## Applications in lymphatic vessel reconstruction

Yang differentiated human adipose-derived stem cells into lymphatic-like endothelial cells, and then the induced cells were seeded into decellularized arterial scaffolds to construct lymphatic vessels. The results showed that the seeded cells proliferated and adhered well in the superficial layer of the decellularized arterial scaffold [94]. However, the study requires further experiments to assess the function of the lymphatic vessel grafts in vivo.

In addition, decellularized vascular matrix can be used for oral mucosa repair [95], bone tissue engineering [96, 97], nerve repair [98], bile duct reconstruction [99], etc.

## Conclusions

The decellularized vascular matrix mainly comprises collagen, elastin, glycosaminoglycans, and other bioactive factors. The collagen and elastin within the matrix ensure the mechanical strength and flexibility of the decellularized vessels. The combined action of collagen, glycosaminoglycans and various active factors contributes to cell adhesion and growth. The reticulated voids of the matrix scaffold provide ample space for cell proliferation and matrix deposition. Combining decellularized vascular scaffolds with other materials to form composite scaffolds, or using EPCs, ECs, VSMCs, stem cells and so on to recellularize the scaffolds in vitro can significantly improve the performance of the scaffolds.

Currently, decellularized vascular scaffolds are mainly used for vascular grafts. After implantation, the scaffolds start to perform their functions such as biocompatibility, remodeling of the lumen and re-endothelialization. Seed cells, scaffold material and cytokine are the three essential elements in tissue engineering [100], among which scaffold material is the key to tissue engineering. Vascular tissues are decellularized to remove natural antigens, while retaining functional ECM and three-dimensional spatial structure of tissues, and biocompatibility and effect of antithrombosis are better than those of synthetic materials. Despite the success of decellularized vascular scaffolds, recellularization of decellularized scaffolds is not yet ideal, and their clinical application needs to be further experimentally explored [101]. At present, it has made some progress in the transplantation of large and medium-sized vessels, but more in vivo studies are still needed in the transplantation of small-diameter vessels, especially studies in large animal models. Main causes of vascular graft failure are related to thrombosis, intimal hyperplasia and vascular calcification. Although scholars have conceived many strategies to improve the performance of decellularized vascular scaffolds, it is still a great challenge to promote the adhesion and fusion of ECs on the lumen of scaffolds, to avoid thrombosis and maintain lumen patency. The combination of decellularized vascular scaffold with other natural biomaterials or synthetic degradable polymers can form a composite scaffold material with superior performance and repair effect, which is expected to be a new type of scaffold material for tissue and organ regeneration and reconstruction. Biomaterials based on decellularized vascular matrix are not only limited to vascular tissue engineering, but also have promising applications in cartilage tissue engineering, urological tissue engineering and other tissue engineering, although their long-term clinical applications and mechanisms of function need to be further investigated.

## Data Availability

Not applicable.

## Abbreviations

EPTFE: Expanded polytetrafluoroethylene
ECM: Extracellular matrix
SDS: Sodium dodecyl sulfate
SD: Sodium deoxycholate
EPCs: Endothelial progenitor cells
VECs: Vascular endothelial cells
VSMCs: Vascular smooth muscle cells
CKC: Carboxymethyl κ carrageenan
S1P: Sphingosine-1-phosphate
bFGF: Basic fibroblast growth factor
VEGF: Vascular endothelial growth factor
GCSF: Granulocyte colony stimulating factor
HG: Hydrogel
PCL: Polycaprolactone
CBP: Collagen binding peptide
CCN1: Matricellular protein 1
HLC-I: Human-like collagen I
ACVM: Acellular vascular matrix
SDF1α: Stromal derived factor 1α
PPF: Polypropylene fumarate
dECM: Decellularized extracellular matrix
GO: Graphene oxide
POC: Poly-octane diol citrate
UV: Ultraviolet ray
MSCs: Mesenchymal stem cells
